# Lord Lister: II. The Lessons of His Early Life

**Published:** 1918-02-02

**Authors:** 


### LORD LISTER.

### II.—The Lessons of his Early Life.

LISTER came of a yeoman stock. He was by descent a Yorkshireman, and belonged to a Quaker family. His great-grandfather, grandfather, and father were business men, and traded in the City of London. Their prosperity increased in each generation, and John Lister, the father of Joseph, afterwards Lord Lister, was able to transmit to his son, one of four children, wealth that, if not beyond the dreams of avarice, was ample to place Joseph Lister in easy circumstances, and not only to keep him during his whole life free from the fear of want and free from the necessity of doing merely pot-boiling work, but also to enable him in early life at the outset of his career to follow his bent, to adopt the profession of his choice, and to follow it in the manner most attractive to him.

There are some characters that need the spur of want to compel them to exert themselves and put forth all their powers. Many and many a man has been ruined by the possession of a competence sufficient to raise him above the need of working for his living. Others there are who are spurred by ambition, and, competence or no competence, will fight their way to the front, set their mark upon their age, and bequeath their names and their memories to posterity. Lister belonged to neither of these. From his Yorkshire ancestry he derived the grit and determination characteristic of the men of that county. From his Quaker upbringing he derived a strong sense of duty, a distaste of frivolity, and an appreciation of the value of time; and besides these, he inherited a sympathetic nature and an originality of mind that made him acutely aware of the sufferings of humanity on the one hand, and on the other gave him a determination to relieve these sufferings as far as lay in his power. He was not a man to fold his hands and sit down resignedly under the conviction that ills are inevitable and must be borne. He was energetic, hardworking, patient, sympathetic, and, above all, he had that quality that is so good a servant and so bad a master, without which no great thing was ever done, but by which misdirected and unregulated so much evil is done, he was enthusiastic. It is delightful to read his own account of his early experiments for the enthusiasm they display.

Such were his moral qualities; and, united as they were in him with a keen intelligence, they promised to carry him far, and they kept their promise. Lister was not what would be called a very clever man. His intellect, though sound, keen and fresh, and of a very high order, was not, perhaps, of the very first order. He succeeded more by the strength of his moral qualities than by reason of his intellectual power; but he had one intellectual quality of the very highest importance. He never lost sight of the end in view. From his earliest introduction into surgery he was impressed with the frightful mortality it entailed, and he would never reconcile himself to the fatalistic view that was then general that this mortality was inescapable and inevitable. It seemed to him that there must be some way of avoiding it, some means by which it could be combated; and to discover this means became the ruling passion of his life. To it he devoted all his energies. In pursuit of his object he plunged into many investigations of many kinds. He pursued some of them very far; but he never lost sight of the main object of pursuit. He kept the ultimate end steadily in view, and this is the main condition of success. This is, if not the highest of intellectual qualities, at any rate the most useful. It is the *sine quâ non* of intellectual effort.

Lister, like nearly all men of exceptional originality of mind, like nearly all men who have made great discoveries or great inventions, owed nothing to public school or university training in early life. As a Quaker, he was naturally sent to schools established by Quakers, managed by Quakers, and supported by Quakers; and whatever is done by Quakers as a body is done efficiently and well. Under the regulations in force at Oxford and Cambridge at the time, Quakers were excluded from these seats of learning, and Lister was at the same time deprived of any advantage they might have been to him, and saved from the sterilising influence of their routine. At the Quaker schools he received a good education, and in early manhood he entered at University College, having chosen medicine, or rather surgery, as the career of his life.

Even when at school his proclivity to science, to biological science, and especially to medical science, showed itself. He prepared skeletons and liked to draw sketches of bones. He put up the skeleton of a frog. He macerated a sheep's head. He wrote an essay on Osteology, and another on the Similarity of Structure between a Monkey and a Man. He had an affectionate and judicious father, who encouraged him in these studies, a very remarkable attitude for a business man at that time to take; and from a very early age Joseph Lister determined that when he grew up he would be a surgeon. The precocious determination calls to mind the instances of Mozart, of Lawrence, of Chatterton, of Pascal, and other men whose strong natural bent was to bring them fame in after-years. From this determination Lister never wavered, and after some misgiving, for no member of the family had ever before entered a profession, his father consented, and the young Lister was entered at University College.

Here he worked very hard for the matriculation at the University of London, and here he had an attack of small-pox. Returning to work too soon, and resuming his habits of great industry, he suffered the natural consequence in what his

☛ The first article appeared January 26.

**LORD LISTER**—(*continued*).

biographer calls, in the elliptic expression of the present day, a serious nervous breakdown. In plain words he had a sharp attack of melancholy, and was for a time in some degree insane. Fortunately for him and his family, Kraspelin was not then born, and therefore had not begun to teach, or Lister would certainly have been pronounced to be suffering from dementia prœcox, and a hopeless diagnosis would have been given. As it was, he was compelled to take a long rest, during which he travelled in Ireland, and at the end of a few months was able to resume his studies, and never again was troubled with this malady. That a man of such great intellectual power and of such great subsequent achievement should have suffered in this way is a lesson and an encouragement to all young men, and they are many, who break down in a similar way from overstudy. Lister was only twenty-one at the time, he was conscientious, enthusiastic, and imprudently overtaxed his strength; and he suffered the natural consequence. The incident shows that not even the greatest minds are exempt from the common lot of humanity; that no mind is so strong that it may not become deranged if it is subjected to a strain greater than it can bear; that such an incident may occur in the life of any man and may yet leave no trace of mental weakness behind it; and that lads who show enthusiasm for their work and are inclined to work too hard, should be carefully supervised by their elders, and warned in time. Lister's case was one of many. He had judicious advisers and ample means, and could afford to take a rest; but others are not so fortunate. Moreover, he was a man, and his sex exempted him from the additional strain that women have to bear at this time of life. Young women who adopt the medical profession are more prone than young men are to take to their studies with indiscreet enthusiasm, and "nervous breakdowns," so-called, are therefore more frequent in them. The case of Joseph Lister should teach them, first, the danger of uncontrolled enthusiasm for their work, and, second, that if they do indulge in uncontrolled enthusiasm and suffer its natural consequence they need not despair. A happy, long, and useful life, even a very distinguished career, may still lie before them.

